# Immune microenvironment and intrinsic subtyping in hormone receptor-positive/HER2-negative breast cancer

**DOI:** 10.1038/s41523-021-00223-x

**Published:** 2021-02-12

**Authors:** Gaia Griguolo, Maria Vittoria Dieci, Laia Paré, Federica Miglietta, Daniele Giulio Generali, Antonio Frassoldati, Luigi Cavanna, Giancarlo Bisagni, Federico Piacentini, Enrico Tagliafico, Katia Cagossi, Guido Ficarra, Aleix Prat, Pierfranco Conte, Valentina Guarneri

**Affiliations:** 1 Department of Surgery, Oncology and Gastroenterology, University of Padova, Padova, Italy; 2 Division of Oncology 2, Istituto Oncologico Veneto IOV—IRCCS, Padova, Italy; 3 Division of Oncology, Hospital Clinic de Barcelona/IDIBAPS, Barcelona, Spain; 4 SOLTI Breast Cancer Research Group, Barcelona, Spain; 5 Multidisciplinary Unit of Breast Pathology, AO. Istituti Ospitalieri di Cremona, Cremona, Italy; 6 Clinical Oncology, Department of Morphology, Surgery and Experimental Medicine, S Anna University Hospital, Ferrara, Italy; 7 Oncology Unit, Department of Oncology and Hematology, Piacenza General Hospital, Piacenza, Italy; 8 Department of Oncology and Advanced Technologies, Oncology Unit, Azienda Unità Sanitaria Locale – IRCCS di Reggio Emilia, Reggio Emilia, Italy; 9 Division of Medical Oncology, Department of Medical and Surgical Sciences for Children and Adults, University Hospital of Modena, Modena, Italy; 10 Center for Genome Research, University of Modena and Reggio Emilia, Modena, Italy; 11 Breast Unit, Ramazzini Hospital, Carpi, Italy; 12 Department of Pathology, Azienda Ospedaliero-Universitaria Policlinico di Modena, Modena, Italy

**Keywords:** Tumour immunology, Tumour heterogeneity, Breast cancer, Tumour heterogeneity, Tumour immunology

## Abstract

Little is known regarding the interaction between immune microenvironment and tumor biology in hormone receptor (HR)+/HER2− breast cancer (BC). We here assess pretreatment gene-expression data from 66 HR+/HER2− early BCs from the LETLOB trial and show that non-luminal tumors (HER2-enriched, Basal-like) present higher tumor-infiltrating lymphocyte levels than luminal tumors. Moreover, significant differences in immune infiltrate composition, assessed by CIBERSORT, were observed: non-luminal tumors showed a more proinflammatory antitumor immune infiltrate composition than luminal ones.

Hormone receptor-positive/HER2-negative (HR+/HER2−) breast cancers (BC) account for almost two-thirds of BC diagnoses^1^ and are well known to be a clinically heterogenous subgroup of BCs. Two methodologies, gene-expression profiling and assessment of immune microenvironment, have significantly impacted our understanding of biological heterogeneity in HR+/HER2− BC. Indeed, non-luminal subtypes by PAM50 (HER2-Enriched [HER2-E] or Basal-like) represent a non-negligible fraction (5–30%) of HR+/HER2− BCs and are associated with specific clinical characteristics, such as reduced endocrine sensitivity, increased chemo sensitivity, and poorer outcome, as compared to Luminal-A or Luminal-B counterparts^2,3^. Regarding the immune microenvironment, HR+/HER2− BCs are generally described as immunologically cold tumors, presenting lower levels of tumor-infiltrating lymphocytes (TILs, as evaluated on H&E stained slides^4^) and PD-L1 (Programmed cell Death protein-Ligand 1)^5–7^. However, around 10% of HR+/HER2− BCs present as immunologically “hot” tumors with ≥60% stromal TILs^5^. Moreover, in a metanalysis including over 1300 HR+/HER2− BC patients treated with neoadjuvant chemotherapy, high TIL levels were associated with significantly higher rates of pathological complete response and paradoxically with significantly shorter overall survival^5^.

In the context of these seemingly conflicting results, assessing the relationship between immune infiltrate levels and composition and tumor biology as assessed by gene-expression profiling in the context of HR+/HER2− BC might be relevant to guide future research. In fact, if increased immune activation (e.g., high TIL levels) in HR+/HER2− BC was associated with more aggressive biological characteristics (such as, endocrine resistance and chemo-sensitivity typical of non-luminal subtypes)^5^, this might lay the basis for the identification of a subset of HR+/HER2− BCs that would be the ideal candidates for testing the combination of immunotherapy and chemotherapy.

We here assess TIL levels and immune infiltrate composition according to intrinsic subtyping in postmenopausal HR+/HER2− BC patients enrolled in a phase-II randomized neoadjuvant trial of letrozole ± lapatinib (LETLOB trial)^8^.

Sixty-six (72%) of 92 patients enrolled in the LETLOB trial had baseline tumor samples meeting quality requirements for gene-expression analysis and were included in the present analysis (REMARK, Supplementary Fig. 1). Characteristics were in line with the overall LETLOB cohort (Supplementary Table 1). All samples included had an ER tumor positivity of at least 30%, while only 15 (23%) had a PgR expression below 10%. All patients included had either grade 2 (51%) or grade 3 (49%) tumors.

Intrinsic subtype distribution, assessed using PAM50 subtype predictor^9^ on 66 pretreatment BC biopsies, was: Luminal-A 39% (*N* = 25), Luminal-B 36% (*N* = 24), Basal-like 18% (*N* = 12), and HER2-E 8% (*N* = 5).

Non-luminal subtypes presented higher Ki67 expression at baseline (median 20% vs 15%, *p* = 0.004) and at surgery (median 10% vs 7%, *p* = 0.002) as compared to luminal subtypes (Supplementary Table 2).

TIL levels were previously evaluated^10^, according to guidelines^11^, on available H&E slides from pretreatment biopsies (*N* = 58) and post-treatment surgery (*N* = 55) samples.

TIL levels significantly differed according to intrinsic subtyping at both timepoints (Fig. 1), with Basal-like tumors showing highest TIL levels and Luminal-A tumors showing lowest TIL levels. Overall, higher TIL levels were observed in non-luminal versus luminal subtypes, both at baseline (*p* = 0.038) and at surgery (*p* = 0.026).

**Fig. 1:**
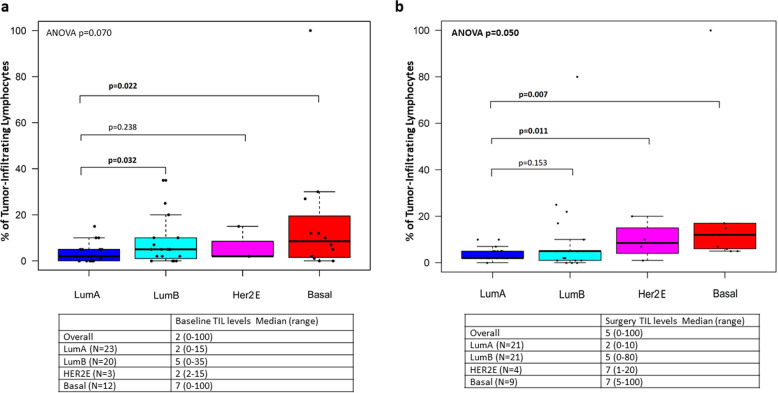
Tumor-infiltrating lymphocyte levels according to intrinsic subtype. **a** Tumor-infiltrating lymphocyte levels at baseline according to intrinsic subtype. **b** Tumor-infiltrating lymphocyte levels at surgery according to intrinsic subtype evaluated on the pretreatment sample. Boxplot legend: centre line: median; bounds of box: interquartile range (IQR); whiskers: highest and lowest value excluding outliers (Q3 + 1.5*IQR to Q1-1.5*IQR); markers beyond the whiskers: potential outliers.

Relative fraction of each immune cell subpopulation was estimated using the CIBERSORT deconvolution method^12^ on gene-expression data from pretreatment samples (64 adequate, two excluded due to poor fitting).

Relative fraction of each immune cell subpopulation in luminal and non-luminal tumors was compared. Non-luminal subtypes presented significantly higher fractions of CD4 memory activated T-cells (*p* = 0.039), follicular helper T-cells (*p* = 0.043), $\gamma$δ T-cells (*p* = 0.009), and M1 macrophages (*p* = 0.001) and lower fractions of T-regulatory cells (*p* = 0.002) and M2 macrophages (*p* = 0.024) than luminal subtypes (Fig. 2a).

**Fig. 2:**
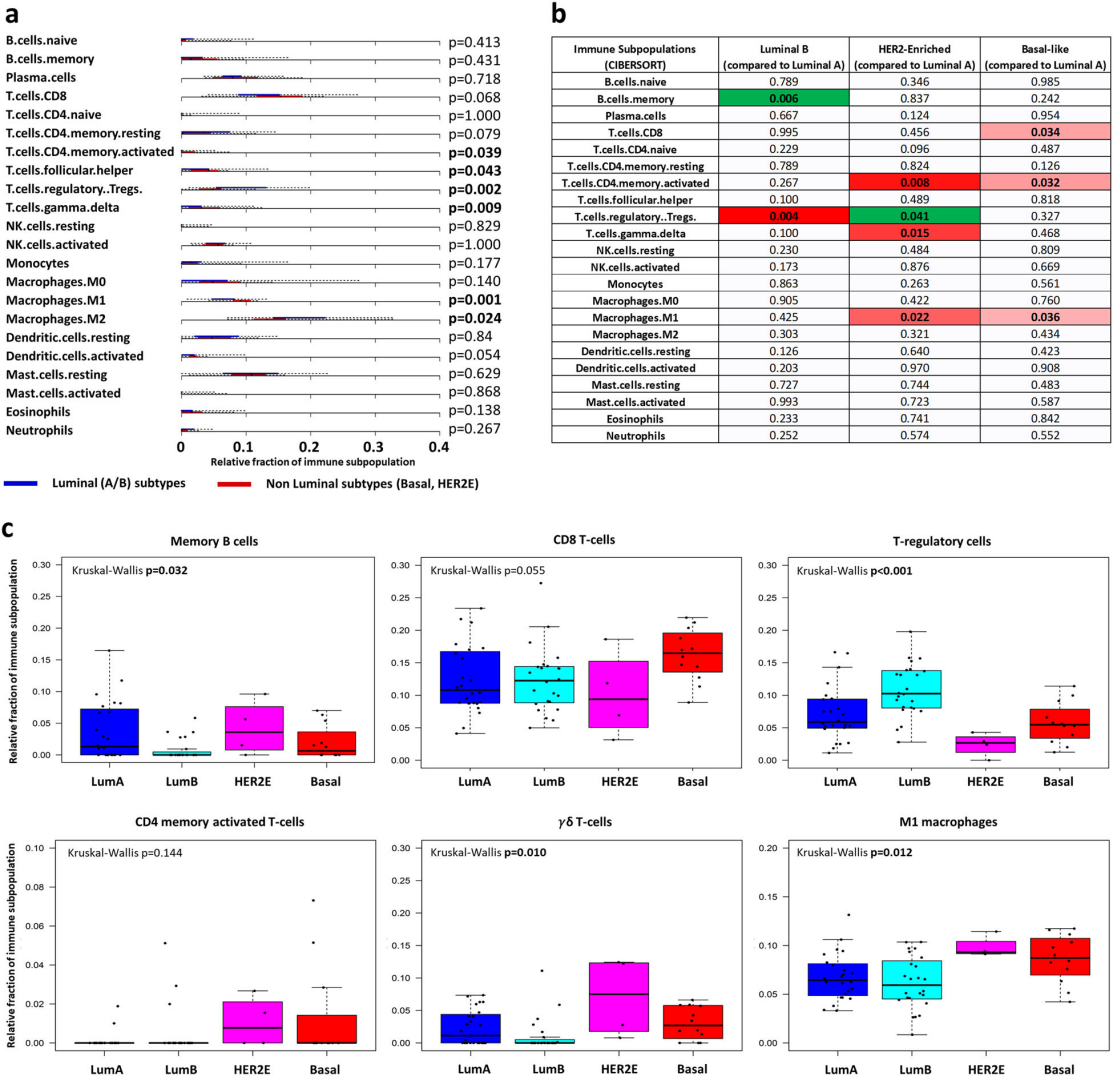
Comparison of relative fraction of each immune cell subpopulation across intrinsic subtypes. **a** Boxplots representing distribution of relative immune cell fraction for each immune cell subpopulation in Luminal (A and B, blue) and non-Luminal (HER2-Enriched and Basal-like, red) tumors. **b** Heatmap showing Mann–Whitney U *p*-values for the comparison of the distribution of immune cell fractions in each intrinsic subtype as compared to Luminal-A tumors (red: higher fraction than in Luminal-A tumors; green: lower fraction than in Luminal-A tumors). **c** Boxplots representing distribution of relative fraction of each immune cell across intrinsic subtypes for immune cell subpopulations presenting significant differences in distribution. Boxplot legend: centre line: median; bounds of box: interquartile range (IQR); whiskers: highest and lowest value excluding outliers (Q3 + 1.5*IQR to Q1-1.5*IQR); markers beyond the whiskers: potential outliers.

Moreover, differences in distribution of relative fraction of each immune cell subpopulation according to each intrinsic subtype were evaluated using Luminal-A subtype as reference (Fig. 2b). Significant differences were observed in relative fraction of Memory B cells (lower in Luminal-B tumors), CD8 T-cells (higher in Basal-like tumors), activated CD4+ Memory T-cells (higher in both HER2-E and Basal-like tumors), Regulatory T-cells (highest in Luminal-B and lowest in HER2-E tumors), and M1 macrophages (higher in HER2-E and Basal-like tumors) (Fig. 2b, c).

Non-luminal subtypes also presented a significantly higher expression of a TGF-β response metagene score (*p* = 0.020) as compared to luminal subtypes (higher in Basal-like and lowest in Luminal-B tumors) (Supplementary Fig. 2a, b).

To assess response to neoadjuvant endocrine treatment (letrozole ± lapatinib), PEPI score was calculated as by published definition^13^ using centrally evaluated Ki67.

A higher PEPI score was significantly associated with higher TIL levels at surgery (Spearman rho = 0.430, *p* = 0.001), but not at baseline (Spearman rho = 0.114, *p* = 0.422), although tumors with PEPI score ≥4 showed numerically higher TIL levels at both timepoints (Supplementary Fig. 3, Supplementary Table 3).

When considering relative fraction of each immune cell subpopulation (Supplementary Table 4–6), a higher fraction of regulatory T-cells (Spearman rho = −0.268, *p* = 0.044) and monocytes (Spearman rho = −0.342, *p* = 0.009) at baseline was associated with lower PEPI scores after neoadjuvant treatment, while a lower relative fraction of M1 macrophages was associated with lower PEPI scores after neoadjuvant treatment (Spearman rho = 0.412, *p* = 0.001).

In this study, assessing 66 HR+/HER2− BC samples from postmenopausal patients enrolled in the LETLOB trial, even though the majority of tumors were classified as Luminal (A or B; 74%), a significant proportion of non-luminal subtypes (26%) was observed. TIL levels varied significantly according to intrinsic subtypes, with basal-like subtype showing highest levels. This observation is consistent with a previous report by Waks et al.^14^ in a smaller group of HR+/HER2− BCs (*N* = 37, *N* = 6 Basal-like), and with the more general observation that higher TIL levels in HR+/HER2− BC are associated with higher Ki67 and lower ER levels^15^, both characteristics associated to non-luminal subtypes in HR+/HER2− BC^3^.

Moreover, we observed significant differences in the composition of immune infiltrate across intrinsic subtypes. Non-luminal subtypes presented significantly higher fractions of CD4 memory activated T-cells, follicular helper T-cells, $\gamma$δ T-cells, and M1 macrophages and lower fractions of T-regulatory cells and M2 macrophages than luminal subtypes, highlighting that higher levels of immune infiltration were also associated with a more proinflammatory antitumour immune infiltrate composition in these non-luminal tumours. Interestingly, previous studies applying CIBERSORT method to publicly available datasets have consistently reported an association between higher M1 fraction in HR+/HER2− early BC and more favorable response to chemotherapy, while conflicting results have been reported on the association with long term outcomes^14,16,17^. In our study, a higher M1 macrophage fraction is associated with less favorable response to endocrine treatment (higher PEPI scores), an effect observed even when considering only luminal PAM50 subtypes (Supplementary Table 6). These HR+/HER2− basal-like inflamed tumors might be the ideal candidates for chemo/immunotherapy trials, similarly to their triple-negative counterparts.

We also identified an association between higher monocyte fraction and higher response to endocrine treatment (lower PEPI score), consistently with previous observations describing more favorable overall survival in HR+ BC with high monocyte fraction^17^.

Interestingly, we also observed significantly higher expression of a TGF-β signaling response signature in non-luminal as compared to luminal HR+ BCs. The present data does not allow to clarify if this is linked to the presence of more immune infiltrate in non-luminal versus luminal subtypes or to a more immunosuppressive immune regulation (TGF− β signaling is generally associated a more immunosuppressive phenotype).

Our exploratory analysis is based on a homogeneous group of HR+/HER2− BC patients included in a clinical trial and represents, to our best knowledge, the largest cohort of HR+/HER2− BC patients for which both PAM50 and CIBERSORT data have been generated. Due to the exploratory nature of the analysis statistical correction for multiplicity was not applied. Moreover, all patients included in the trial were postmenopausal, therefore if these results apply to HR+/HER2− BC in premenopausal women remains unknown.

Despite these limitations, this study highlights the relevant interactions between tumor biology and immune microenvironment in HR+/HER2− early BC, which should be kept in mind when analyzing the role of immunity and planning immunotherapy-based clinical trials in this BC subtype.

## Methods

### Patients

The LETLOB trial (NCT00422903, first posted on clinicaltrials.gov January 17, 2007) is a multicenter, phase-II trial which randomized 92 HR+/HER2− postmenopausal BC patients (stage II-IIIA; T > 2 cm, N0-1, M0) to receive letrozole 2.5 mg daily plus lapatinib 1500 mg daily (arm A) or placebo (arm B) for 6 months. Previously published study results have shown that the combination of letrozole–lapatinib is feasible and results in similar overall clinical response rate and effect on Ki67 as compared to letrozole-placebo^8^.

The trial was approved by the relevant ethics committees (Comitato Etico Provinciale di Modena) and patients provided written informed consent. All procedures performed in studies involving human participants were in accordance with the ethical standards of the institutional and/or national research committee and with the 1964 Helsinki declaration and its later amendments or comparable ethical standards.

As part of the original study protocol, pretreatment frozen core biopsies and FFPE tumor samples from both diagnostic core biopsies and surgical samples were collected.

### TIL, PEPI, and gene expression

TIL levels were centrally evaluated following consensus guidelines^4^ on H&E stained slides from both diagnostic core biopsies and surgical, as previously published^10^. PEPI score was calculated as by published definition^13^.

As part of the original study protocol, RNA was extracted from pretreatment frozen core biopsies using the commercial kit RNeasy Mini Kit (Qiagen, Valencia, CA, USA) and total RNA was quantified using the NanoDrop ND-1000 spectrophotometer (Thermo Fisher Scientific, Freemont, CA, USA). RNA quality was assessed by evaluating the A260/A280 and A260/230 ratios of each sample and by means of capillary electrophoresis using the Agilent 2100 Bioanalyzer with the RNA 6000 Nano Assay kit (Agilent Technologies, Palo Alto, CA, USA). Samples that met quality requirements were further processed according to the Affymetrix GeneChip® 3′ IVT Express Kit user’s manual, starting from 150 ng of total RNA for each sample, as previously reported^8^. Adequate gene-expression data were available for 66 out of 92 patients enrolled.

### PAM50, CIBERSORT, and TGF-beta signature

Probe level data were normalized and converted to expression values using robust multiarray average (RMA) procedure. Quality control assessment was performed in R statistical environment using affy, affyQCReport, and affyPLM Bioconductor packages.

PAM50 subtype predictor was used to assign intrinsic subtype using nearest centroid procedure^9^. If the nearest centroid for a sample was Normal-like, second nearest centroid was selected.

Proportion of infiltrating immune cell subsets was calculated using the CIBERSORT deconvolution method (leukocyte gene signature matrix LM22, 500 permutations setting)^12^. A 0.05 *p*-value threshold for the deconvolution result was used to filter out samples with poor fitting.

A previously published TGF-β signaling response gene-expression signature^18^ was calculated and compared between luminal and non-luminal subtypes.

### Statistical analysis

Distribution of clinical/pathological characteristics between subgroups was compared using Chi-Square test, Fisher’s Exact test, or Student *t*-test according to type of variable analyzed.

Associations between TIL levels and relative fraction of immune cell subtypes and qualitative variables were determined by Student *t-* and ANOVA or Mann–Whitney U and Kruskal–Wallis tests, respectively. Spearman correlation was used to correlate these variables and PEPI score. All statistical tests were two-sided, considered significant when *p* < 0.05 and conducted using R software (version 3.6.2)^19^.

### Reporting summary

Further information on research design is available in the Nature Research Reporting Summary linked to this article.

## Supplementary information

Supplemental Material

Reporting Summary Checklist

## Data Availability

The datasets that support the findings of this study are not publicly available in order to protect patient privacy. The data will be available on reasonable request from the corresponding author, M.V.D., email address: mariavittoria.dieci@unipd.it. The data generated and analysed during this study are described in the following metadata record: 10.6084/m9.figshare.13516520^20^.

## Code availability

The R software (version 3.6.2) codes used during the current study are available from the corresponding author on reasonable request.
