# Supplementary material for: Immune microenvironment and intrinsic subtyping in hormone receptor-positive/HER2-negative breast cancer
Source: NPJ Breast Cancer. 2021 Feb 12;7:12. doi: 10.1038/s41523-021-00223-x (PMC7881184; doi:10.1038/s41523-021-00223-x)
Supplement: Supplementary file 1 — Supplemental Material [file 41523_2021_223_MOESM1_ESM.pdf]

Supplementary Material

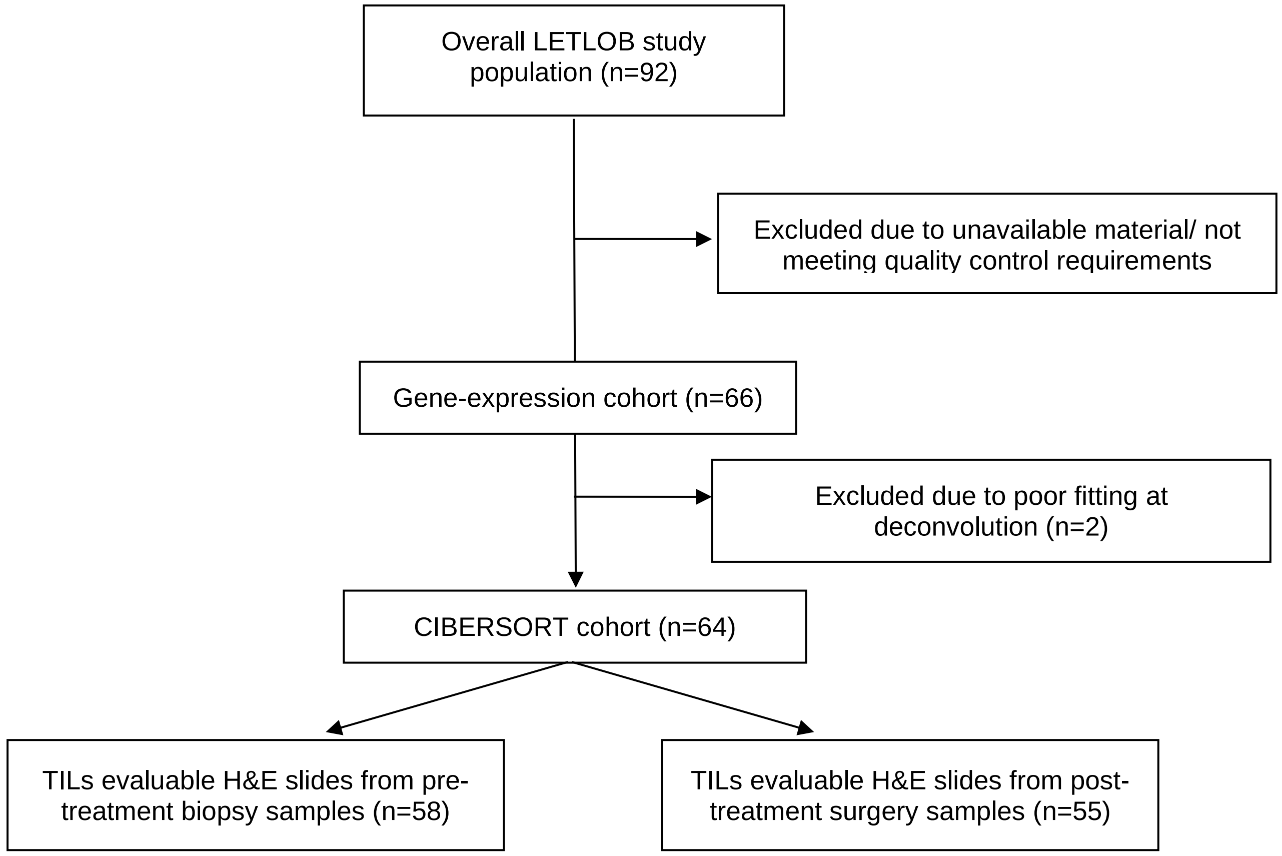

Supplementary Figure 1. REMARK diagram. Derivation of the analyzed cohort from the original LETLOB trial cohort.

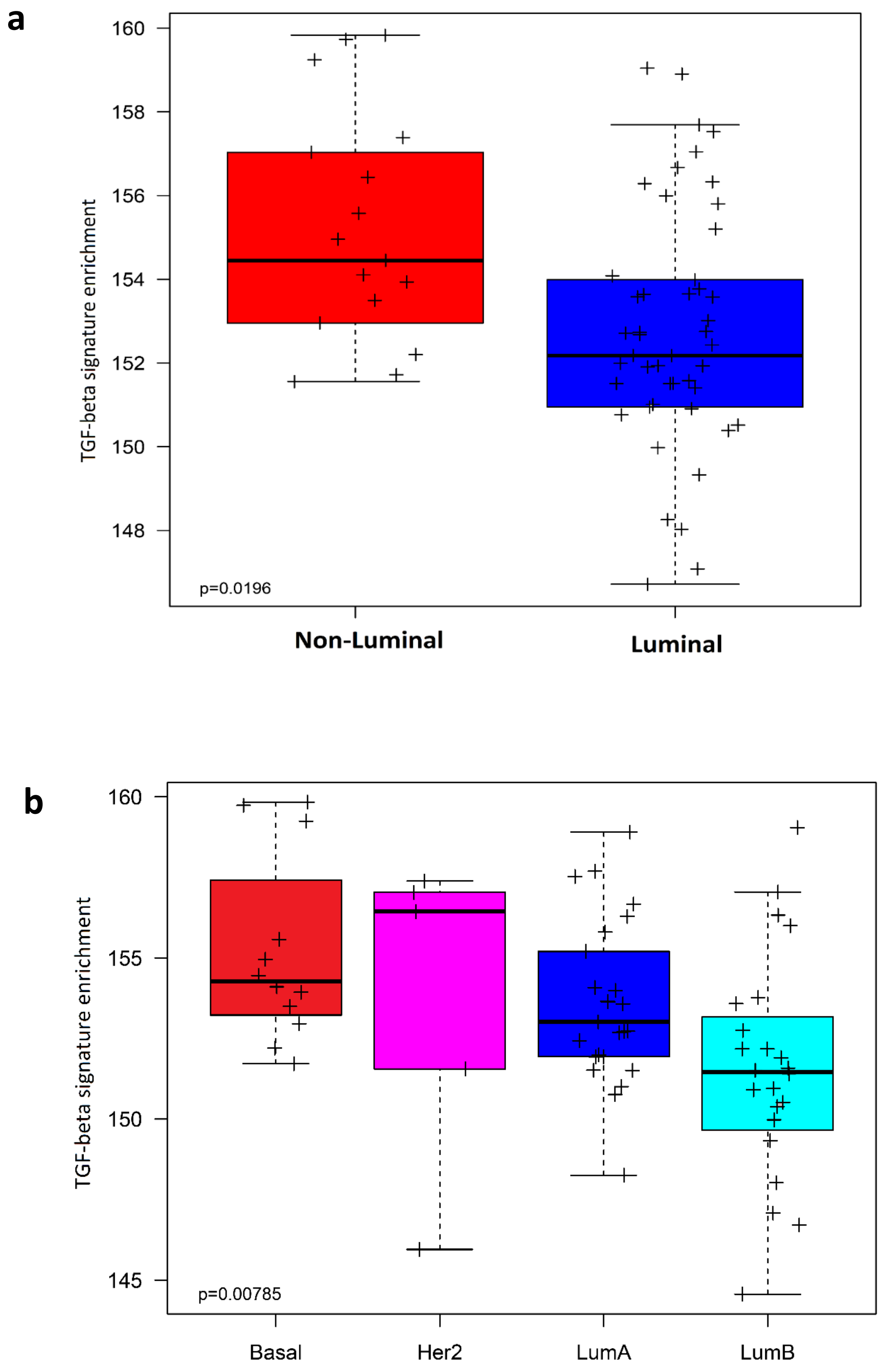

Supplementary Figure 2. TGF-beta gene expression signature score according to intrinsic subtyping.
a. TGF-beta gene expression signature score in Luminal (Luminal A and Luminal B, blue) and non-Luminal (HER2-Enriched and Basal-like, red) tumors.
b. TGF-beta gene expression signature score according to PAM50 intrinsic subtype.
Boxplot legend: centre line: median; bounds of box: interquartile range (IQR); whiskers: highest and lowest value excluding outliers (Q3+1.5*IQR to Q1-1.5*IQR; markers beyond the whiskers: potential outliers.

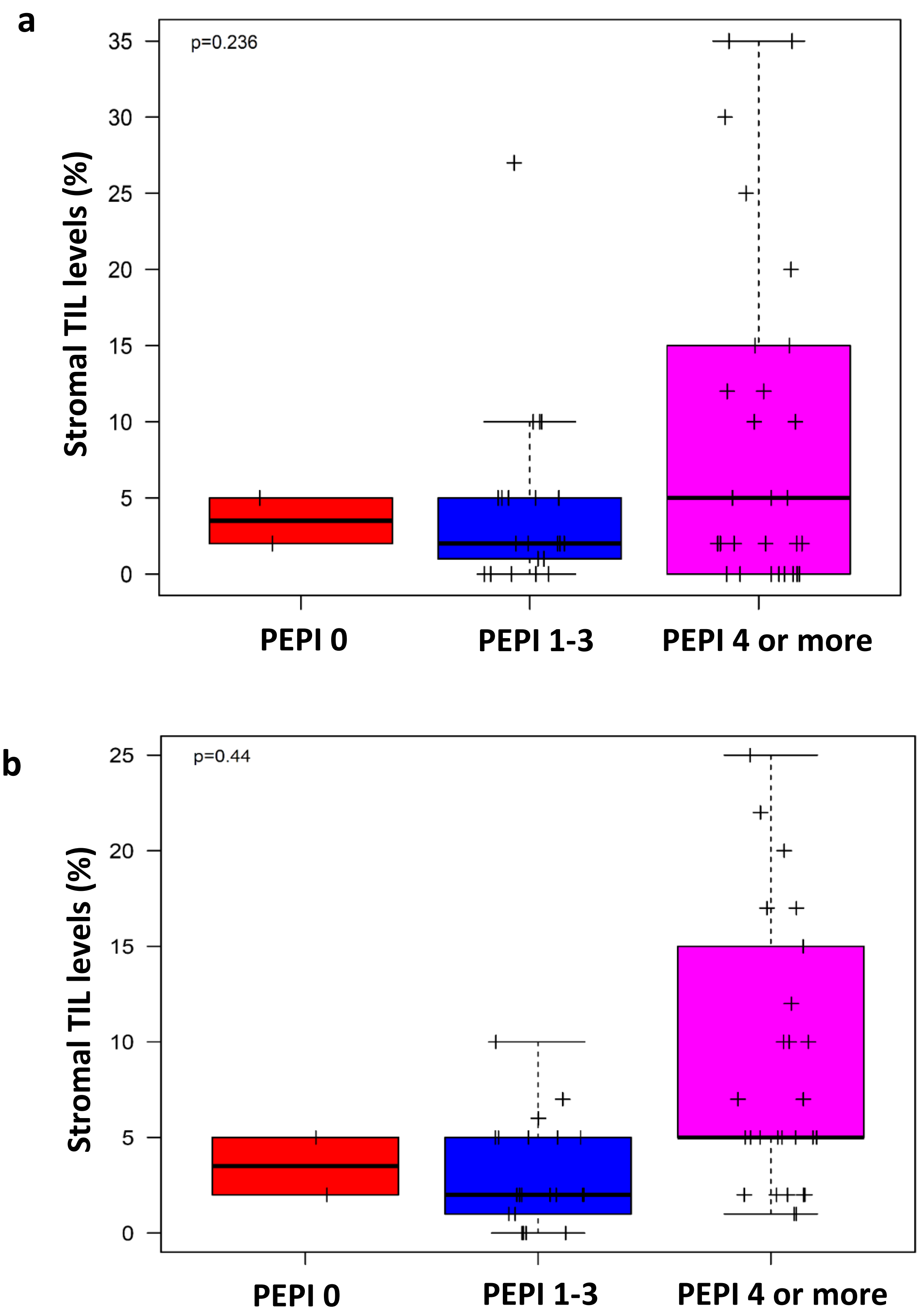

Supplementary Figure 3. TIL levels according to PEPI score

a. TIL levels at baseline according to PEPI score

b. TIL levels at surgery according to PEPI score

Boxplot legend: centre line: median; bounds of box: interquartile range (IQR); whiskers: highest and lowest value excluding outliers (Q3+1.5*IQR to Q1-1.5*IQR; markers beyond the whiskers: potential outliers.

Supplementary Table 1. Comparison between baseline patient and disease characteristics in the overall LETLOB study cohort and in the present study cohort

Variable		LETLOB cohort (N=92)	Present cohort (N=66)	p-value
Age in years, median (range)		70 (47-88)	68 (47-87)	0.476
Median ER expression % (range)		95 (30-100)	95 (30-100)	0.244
Median PgR expression % (range)		70 (0-100)	68 (0-100)	0.952
Median baseline Ki67 expression % (range)		17 (2-60)	15 (2-60)	0.553
Median post-treatment Ki67 expression % (range)		10 (1-50)	9 (1-50)	0.735
Median baseline TIL% (range)		2 (0-100)	2 (0-100)	1
Median post-treatment TIL% (range)		5 (0-100)	5 (0-100)	1
		**N (%)**	**N (%)**	
Clinical stage	IIA	47 (51%)	35 (53%)	0.922
	IIB	38 (41%)	27 (41%)	
	IIIA	7 (8%)	4 (6%)	
Histology	Ductal	67 (73%)	50 (76%)	0.878
	Lobular/other/NA	25 (27%)	16 (24%)	
Histologic Grade	G1	2 (3%)	0 (0%)	0.533
	G2	35 (51%)	28 (51%)	
	G3	32 (47%)	27 (49%)	
Treatment arm	Lapatinib	43 (47%)	28 (42%)	0.591
	Placebo	49 (53%)	38 (58%)	

Supplementary Table 2. Comparison between demographic and disease characteristics of patients whose tumor was characterized as Luminal (Luminal A or Luminal B) or Non-Luminal (HER2E or Basal-like) according to PAM50

Variable		Luminal A/B (N=49)	Non-Luminal (N=17)	p-value
Age in years, median (range)		69 (47-87)	63 (52-81)	0.344
Median ER expression % (range)		95 (50-100)	95 (30-100)	0.318
Median PgR expression % (range)		65 (0-100)	60 (0-100)	0.669
Median baseline Ki67 expression % (range)		15 (2-48)	20 (2-60)	**0.004**
Median post-treatment Ki67 expression % (range)		7 (1-35)	10 (3-50)	**0.002**
Median baseline TIL% (range)		2 (0-35)	7 (0-100)	**0.038**
Median post-treatment TIL% (range)		2 (0-80)	7 (1-100)	**0.026**
		**N (%)**	**N (%)**	
Clinical stage	IIA	29 (59%)	6 (35%)	0.164
	IIB	17 (35%)	10 (59%)	
	IIIA	3 (6%)	1 (6%)	
Histology	Ductal	36 (74%)	14 (82%)	0.652
	Lobular/other/NA	13 (26%)	3 (18%)	
Histologic Grade	G1	0 (0%)	0 (0%)	0.058
	G2	24 (60%)	4 (27%)	
	G3	16 (40%)	11 (73%)	
Treatment arm	Lapatinib	21 (43%)	7 (41%)	1
	Placebo	28 (57%)	10 (59%)	

Supplementary Table 3. Correlation between TILs and PEPI score in different subgroups based on intrinsic subtyping and treatment

	Correlation between pre-treatment TILs and PEPI score		Correlation between post-treatment TILs and PEPI score	
	**Spearman rho**	**p-value**	**Spearman rho**	**p-value**
**Treatment Letrozole-Placebo (N=38)**	0.1161176	0.5339	0.4827735	**0.004433**
**Treatment Letrozole-Lapatinib (N=28)**	0.13446	0.5612	0.4561224	**0.04324**
**PAM50 subtype non luminal (N=17)**	0.1815163	0.5346	0.7881364	**0.0014**
**PAM50 subtype luminal (N=49)**	-0.02379795	0.8872	0.236488	0.1418

Supplementary Table 4. Correlation between relative fraction of each immune subpopulation and PEPI score

Immune Subpopulations (CIBERSORT)	Correlation between relative fraction of immune subpopulation and PEPI score	
	Spearman rho	p value
B.cells.naive	-0.1806436	0.1787
B.cells.memory	0.03416989	0.8008
Plasma.cells	0.1391593	0.3019
T.cells.CD8	0.1158989	0.3906
T.cells.CD4.naive	-0.0338259	0.8027
T.cells.CD4.memory.resting	-0.1600183	0.2344
T.cells.CD4.memory.activated	0.2281466	0.08784
T.cells.follicular.helper	0.1181889	0.3812
T.cells.regulatory..Tregs.	**-0.2680813**	**0.04378**
T.cells.gamma.delta	0.2373358	0.07546
NK.cells.resting	0.1409412	0.2957
NK.cells.activated	0.14651	0.2768
Monocytes	**-0.3424303**	**0.009124**
Macrophages.M0	0.1445119	0.2835
Macrophages.M1	**0.4120068**	**0.001451**
Macrophages.M2	-0.009465248	0.9443
Dendritic.cells.resting	-0.05202587	0.7007
Dendritic.cells.activated	0.04902599	0.7172
Mast.cells.resting	0.01775573	0.8957
Mast.cells.activated	0.008836042	0.948
Eosinophils	-0.09196236	0.4963
Neutrophils	-0.07167551	0.5962

Supplementary Table 5. Correlation between relative fraction of each immune subpopulation and PEPI score according to treatment

Immune Subpopulations (CIBERSORT)	Correlation between relative fraction of immune subpopulation and PEPI score			
	Treatment Letrozole-Placebo (N=38)		Treatment Letrozole-Lapatinib (N=26)	
	Spearman rho	p-value	Spearman rho	p-value
**B.cells.naive**	-0.1144889	0.5191	-0.2782855	0.1985
**B.cells.memory**	-0.083125	0.6402	0.2120927	0.3313
**Plasma.cells**	0.2002573	0.2561	0.1328992	0.5455
**T.cells.CD8**	0.1499942	0.3972	0.06491614	0.7685
**T.cells.CD4.naive**	-0.06884659	0.6988	NA	NA
**T.cells.CD4.memory.resting**	-0.1247753	0.4820	-0.2378164	0.2745
**T.cells.CD4.memory.activated**	0.1651265	0.3507	0.3334658	0.12
**T.cells.follicular.helper**	-0.03520519	0.8433	0.381565	0.07241
**T.cells.regulatory..Tregs.**	-0.2058244	0.2429	-0.3542275	0.09725
**T.cells.gamma.delta**	0.08527457	0.6316	**0.467217**	**0.02459**
**NK.cells.resting**	**0.3458814**	**0.0451**	-0.2493923	0.2511
**NK.cells.activated**	0.1536526	0.3856	0.1334103	0.5439
**Monocytes**	-0.2984062	0.08649	-0.4126506	0.05036
**Macrophages.M0**	0.09121467	0.6079	0.2397672	0.2705
**Macrophages.M1**	0.2785151	0.1107	**0.6328046**	**0.001193**
**Macrophages.M2**	-0.05392155	0.762	0.06031579	0.7846
**Dendritic.cells.resting**	0.1051023	0.5541	-0.3115988	0.1478
**Dendritic.cells.activated**	0.01977127	0.9116	0.1366429	0.5341
**Mast.cells.resting**	-0.05805712	0.7443	0.1937261	0.3758
**Mast.cells.activated**	0.01705265	0.9237	-0.04331279	0.8444
**Eosinophils**	0.0650404	0.7148	-0.3286432	0.1257
**Neutrophils**	-0.1215582	0.4934	-0.003861245	0.986

Supplementary Table 6. Correlation between relative fraction of each immune subpopulation and PEPI score according to PAM50 intrinsic subtyping

Immune Subpopulations (CIBERSORT)	Correlation between relative fraction of immune subpopulation and PEPI score			
	PAM50 subtype non-luminal (N=16)		PAM50 subtype luminal (N=48)	
	Spearman rho	p-value	Spearman rho	p-value
**B.cells.naive**	-0.1847435	0.5098	-0.1037614	0.5132
**B.cells.memory**	-0.1635507	0.5603	-0.003197786	0.984
**Plasma.cells**	0.4093923	0.1297	0.06068938	0.7026
**T.cells.CD8**	-0.3738742	0.1698	0.1323824	0.4033
**T.cells.CD4.naive**	-0.1618923	0.5643	-0.0384149	0.8091
**T.cells.CD4.memory.resting**	-0.04335078	0.8781	-0.1055645	0.5058
**T.cells.CD4.memory.activated**	0.2314858	0.4065	0.07328028	0.6447
**T.cells.follicular.helper**	0.2909471	0.2928	-0.03663712	0.8178
**T.cells.regulatory..Tregs.**	-0.347703	0.2041	-0.04536891	0.7754
**T.cells.gamma.delta**	0.2307583	0.408	0.05748884	0.7176
**NK.cells.resting**	0.1618923	0.5643	0.07946723	0.6169
**NK.cells.activated**	0.2691894	0.332	0.1214634	0.4435
**Monocytes**	-0.4116064	0.1274	-0.296626	0.05645
**Macrophages.M0**	**0.5421176**	**0.03683**	-0.1130898	0.4758
**Macrophages.M1**	0.06916673	0.8065	**0.4020565**	**0.008305**
**Macrophages.M2**	-0.01869371	0.9473	0.1954418	0.2148
**Dendritic.cells.resting**	0.1088128	0.6995	-0.1296038	0.4133
**Dendritic.cells.activated**	0.1439416	0.6088	-0.0795611	0.6165
**Mast.cells.resting**	-0.2149777	0.4416	0.06551406	0.6802
**Mast.cells.activated**	0.006319628	0.9822	0.1062567	0.503
**Eosinophils**	-0.2896017	0.2951	-0.08383968	0.5976
**Neutrophils**	0.1054468	0.7084	-0.03781532	0.8121
