# Supplementary material for: Immune microenvironment and intrinsic subtyping in hormone receptor-positive/HER2-negative breast cancer
Source: NPJ Breast Cancer. 2021 Feb 12;7:12. doi: 10.1038/s41523-021-00223-x (PMC7881184; doi:10.1038/s41523-021-00223-x)
Supplement: Supplementary file 2 — Reporting Summary Checklist [file 41523_2021_223_MOESM2_ESM.pdf]

nature research

Corresponding author(s): Maria Vittoria Dieci

Last updated by author(s): Dec 27, 2020

Reporting Summary

Nature Research wishes to improve the reproducibility of the work that we publish. This form provides structure for consistency and transparency in reporting. For further information on Nature Research policies, see our Editorial Policies and the Editorial Policy Checklist.

Statistics

For all statistical analyses, confirm that the following items are present in the figure legend, table legend, main text, or Methods section.

n/a	Confirmed	
☐	☒	The exact sample size (n) for each experimental group/condition, given as a discrete number and unit of measurement
☒	☐	A statement on whether measurements were taken from distinct samples or whether the same sample was measured repeatedly
☐	☒	The statistical test(s) used AND whether they are one- or two-sided<br>*Only common tests should be described solely by name; describe more complex techniques in the Methods section.*
☐	☒	A description of all covariates tested
☒	☐	A description of any assumptions or corrections, such as tests of normality and adjustment for multiple comparisons
☐	☒	A full description of the statistical parameters including central tendency (e.g. means) or other basic estimates (e.g. regression coefficient) AND variation (e.g. standard deviation) or associated estimates of uncertainty (e.g. confidence intervals)
☐	☒	For null hypothesis testing, the test statistic (e.g. F, t, r) with confidence intervals, effect sizes, degrees of freedom and P value noted<br>*Give P values as exact values whenever suitable.*
☒	☐	For Bayesian analysis, information on the choice of priors and Markov chain Monte Carlo settings
☒	☐	For hierarchical and complex designs, identification of the appropriate level for tests and full reporting of outcomes
☐	☒	Estimates of effect sizes (e.g. Cohen's d, Pearson's r), indicating how they were calculated

Our web collection on statistics for biologists contains articles on many of the points above.

Software and code

Policy information about availability of computer code

Data collection	R software (version 3.6.2)
Data analysis	R software (version 3.6.2)

For manuscripts utilizing custom algorithms or software that are central to the research but not yet described in published literature, software must be made available to editors and reviewers. We strongly encourage code deposition in a community repository (e.g. GitHub). See the Nature Research guidelines for submitting code & software for further information.

Data

Policy information about availability of data

All manuscripts must include a data availability statement. This statement should provide the following information, where applicable:

- Accession codes, unique identifiers, or web links for publicly available datasets
- A list of figures that have associated raw data
- A description of any restrictions on data availability

The datasets analysed during the current study are available from the corresponding author on reasonable request

Field-specific reporting

Please select the one below that is the best fit for your research. If you are not sure, read the appropriate sections before making your selection.

☒ Life sciences ☐ Behavioural & social sciences ☐ Ecological, evolutionary & environmental sciences

For a reference copy of the document with all sections, see nature.com/documents/nr-reporting-summary-flat.pdf

Life sciences study design

All studies must disclose on these points even when the disclosure is negative.

Sample size	No sample size calculation performed. Exploratory analysis on biological samples from a clinical trial.
Data exclusions	Adequate gene-expression data was available for 66 out of 92 patients enrolled.
Replication	No replication. Data from biological samples from a clinical trial.
Randomization	Not relevant for the present study. Data from biological samples from a clinical trial reported in this manuscript.
Blinding	Not relevant for the present study. Data from biological samples from a clinical trial reported in this manuscript.

Reporting for specific materials, systems and methods

We require information from authors about some types of materials, experimental systems and methods used in many studies. Here, indicate whether each material, system or method listed is relevant to your study. If you are not sure if a list item applies to your research, read the appropriate section before selecting a response.

Materials & experimental systems

n/a	Involved in the study	
☒	☐	Antibodies
☒	☐	Eukaryotic cell lines
☒	☐	Palaeontology and archaeology
☒	☐	Animals and other organisms
☐	☒	Human research participants
☐	☒	Clinical data
☒	☐	Dual use research of concern

Methods

n/a	Involved in the study	
☒	☐	ChIP-seq
☒	☐	Flow cytometry
☒	☐	MRI-based neuroimaging

Human research participants

Policy information about studies involving human research participants

Population characteristics	The LETLOB trial enrolled HR+/HER2- postmenopausal BC patients (stage II-IIIA; T>2 cm, N0-1, M0).<br>Patient characteristics are detailed in Supplementary Table 1.
Recruitment	The LETLOB trial enrolled HR+/HER2- postmenopausal BC patients (stage II-IIIA; T>2 cm, N0-1, M0).<br>Patient characteristics are detailed in Supplementary Table 1.
Ethics oversight	The study protocol was primarily approved by the Comitato Etico Provinciale di Modena (secondary approval was also give by the Ethics Committees of the participating centers)

Note that full information on the approval of the study protocol must also be provided in the manuscript.

Clinical data

Policy information about clinical studies

All manuscripts should comply with the ICMJE guidelines for publication of clinical research and a completed CONSORT checklist must be included with all submissions.

Clinical trial registration	NCT00422903
Study protocol	The study protocol is available as supplementary material to previous publication (Guarneri V. et al, J Clin Oncol 2014)
Data collection	The collection of clinical data, not relevant to the present study, has been previously published in detail (Guarneri V. et al, J Clin Oncol 2014)

Outcomes

Clinical outcomes, not relevant to the present study, has been previously published in detail (Guarneri V. et al, J Clin Oncol 2014)
